# The Reproducibility and Relative Validity of a Mexican Diet Quality Index (ICDMx) for the Assessment of the Habitual Diet of Adults

**DOI:** 10.3390/nu8090516

**Published:** 2016-08-23

**Authors:** Gabriela Macedo-Ojeda, Fabiola Márquez-Sandoval, Joan Fernández-Ballart, Barbara Vizmanos

**Affiliations:** 1 Academic group “Food and nutrition in health-disease process”, Department of Human Reproduction, Growth and Child Development, Center of Health Sciences (CUCS), University of Guadalajara (UdG), Guadalajara, Jalisco 44100, Mexico; gaby_macedo@yahoo.com.mx (G.M.-O.); fabiola_msandoval@yahoo.com.mx (F.M.-S.); 2 Department of Public Health, Center of Health Sciences (CUCS), University of Guadalajara (UdG), Guadalajara, Jalisco 44100, Mexico; 3 Preventive Medicine and Public Health, IISPV, Universitat Rovira i Virgili, Reus 43201, Spain; joan.fernandez-ballart@urv.cat; 4 CIBER Pathophysiology of Obesity and Nutrition (CB06/03) Instituto Carlos III, Madrid 28029, Spain

**Keywords:** diet questionnaire, diet index, healthy diet, reproducibility, validity

## Abstract

The study of diet quality in a population provides information for the development of programs to improve nutritional status through better directed actions. The aim of this study was to assess the reproducibility and relative validity of a Mexican Diet Quality Index (ICDMx) for the assessment of the habitual diet of adults. The ICDMx was designed to assess the characteristics of a healthy diet using a validated semi-quantitative food frequency questionnaire (FFQ-Mx). Reproducibility was determined by comparing 2 ICDMx based on FFQs (one-year interval). Relative validity was assessed by comparing the ICDMx (2nd FFQ) with that estimated based on the intake averages from dietary records (nine days). The questionnaires were answered by 97 adults (mean age in years = 27.5, SD = 12.6). Pearson (*r*) and intraclass correlations (ICC) were calculated; Bland-Altman plots, Cohen’s κ coefficients and blood lipid determinations complemented the analysis. Additional analysis compared ICDMx scores with nutrients derived from dietary records, using a Pearson correlation. These nutrient intakes were transformed logarithmically to improve normality (log10) and adjusted according to energy, prior to analyses. The ICDMx obtained ICC reproducibility values ranged from 0.33 to 0.87 (23/24 items with significant correlations; mean = 0.63), while relative validity ranged from 0.26 to 0.79 (mean = 0.45). Bland-Altman plots showed a high level of agreement between methods. ICDMx scores were inversely correlated (*p* < 0.05) with total blood cholesterol (*r* = −0.33) and triglycerides (*r* = −0.22). ICDMx (as calculated from FFQs and DRs) obtained positive correlations with fiber, magnesium, potassium, retinol, thiamin, riboflavin, pyridoxine, and folate. The ICDMx obtained acceptable levels of reproducibility and relative validity in this population. It can be useful for population nutritional surveillance and to assess the changes resulting from the implementation of nutritional interventions.

## 1. Introduction

Dietary risks are considered the main factor (from 79 analyzed) to explain deaths and Disability-Adjusted Life-Years (DALYs) in the world [1]. Based on the results of the Global Burden of Disease Study, in 2013, they accounted for about 11.2 (UI-uncertainty interval, 9.6–12.9) million deaths, and 241.3 (UI 209.6–273.3) million DALYs. Among the main dietary risks are low intake of fruit, high sodium, low whole grains, low vegetables, and low nuts and seeds. Furthermore, the analysis of a population’s diet is of prime importance in the field of public health as it contributes to the development of programs to improve it. It also plays an important role in the establishment of policies for the fortification or labeling of food and the determination of nutritional recommendations [2]. 

Diet quality has been defined using various criteria. Previous studies have focused on meeting specific consumption requirements of nutrients or food groups [3,4,5,6] as well as adherence to national or international recommendations regarding habitual diet [7,8,9,10]. Different quality indexes of diet have been constructed: some of them are based on food consumption and could have an immediate interpretation; there are others, nutrient based, that need a previous analysis from food intake, in order to calculate the index in a second moment.

In Mexico, national food guidelines define a healthy diet [11] as one with the following characteristics: (1) Sufficient in the sense that it is enough to meet nutritional needs; (2) Balanced in terms of the proportions of nutrients of which it is made up; (3) Complete, in the sense that it includes foods from these three groups: (a) vegetables and fruits, (b) cereals, (c) legumes and animal products; (4) Varied, in the sense that it includes different foods from each group; (5) Innocuous, in the sense that its regular consumption poses no health risks; (6) Appropriate, in terms of the tastes and culture of its consumers as well as its affordability. 

A previous study on the Mexican population provided a cross-cutting assessment of variation in three dietary indices (food groups and nutrients based) by socio-demographic and anthropometric variables: a Cardioprotective Index (including consumption of fat, fiber, vegetables and fruits), a Micronutrient Adequacy Index (corresponding to micronutrients intake) and a Dietary Diversity Index (among 30 food groups) [12]. All three indices identify aspects of dietary quality which point to nutrients or foods that are not adequately consumed by the population. However, their findings indicate a single index is insufficient to assess all relevant characteristics of a healthy diet and suggest applying all three indices of dietary quality to gain a more complete picture.

The usefulness of a dietary quality index can be assessed in several ways. It could be accomplished through an evaluation of its internal consistency [13], of its association with social or anthropometric constructs [12], with risk factors for disease [9,14,15] or with mortality [16], or by measuring its reproducibility or validity [5,17]. The reproducibility analysis provides information on the ability to measure dietary quality at two specific times to assess their degree of similarity. Validity tests measure the degree of correlation or agreement with a reference method or with biochemical health indicators.

The purpose of this study is to evaluate the reproducibility (with a one-year interval) and relative validity, by comparison with a reference method and with biochemical health indicators of a Mexican Diet Quality Index (ICDMx) based on nutrients and food groups intake, designed to assess five characteristics of a healthy diet [11] using a validated semi-quantitative food frequency questionnaire (FFQ-Mx) [18].

## 2. Materials and Methods

### 2.1. Study Population and Design

Women and men over the age of 18 were recruited through verbal or written requests, provided they met the following inclusion criteria: Mexicans with self-perceived good health who can read and write Spanish. Pregnant or lactating women would not be included, however there was no woman in this case. We excluded those who at the end of the study did not complete all of the dietary questionnaires that were administered. According to Walter Willett [19], the number of suggested subjects for a validation study would be approximately 110, based on the standard one-sample formula for sample size using Fisher’s Z transformation of correlation coefficients.

We have included 150 subjects, at baseline, considering the possible losses to follow up. During the year, 35% did not complete the RD and the FFQ: 21 participants did not come to bring back their first Dietary Register (DR), 17 did not send or bring their second DR and 12 did not send their third DR. Two subjects did not come to the visit for the FFQ2. Another subject was removed because two of his DRs were identical.

This study was conducted in accordance with guidelines laid down in the Declaration of Helsinki, and all procedures involving human subjects were approved by the Ethics Committee of the Center of Health Sciences, University of Guadalajara. Written consent was obtained from all subjects. 

At the start of the study, nutrition professionals collected anthropometric measurements (weight and height) as well as data on the marital status, occupation, and physical activity of participants. They also administered, through a personal interview, a validated FFQ for adults that included 162 food items that were selected to describe usual dietary intake over the past year. Participants were asked to describe their average intake of each food by using nine frequency of consumption categories ranging from “never or almost never” to “more than six times per day”. For each food item, there was a defined portion size in order to adequate the frequency of the consumption. The reproducibility and relative validity of nutrient intakes and food intakes of the FFQ-Mx have been described elsewhere [18]. 

**Figure 1:**
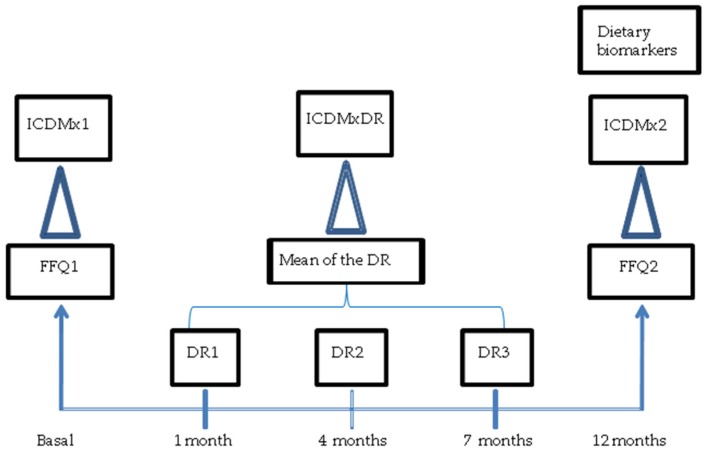
Data recollecting methodology. ICDMx, *Índice de Calidad de la Dieta Mexicana*; FFQ, semi-quantitative food frequency questionnaire; DRs, dietary records; FFQ1, basal FFQ; FFQ2, second FFQ (one year later); ICDMx 1, ICDMx from basal FFQ; ICDMx2, ICDMx from second FFQ (one year latter); ICDMxDR, ICDMx from DRs.

During that same year, we collected dietary records (DRs) from three days each, at three different times, for a total of nine days of assessment. On each occasion, food intake from two working days and one weekend day were included. Participants were previously trained to complete the DRs and had a visual guide to be used for the quantification of food. The correct completion of the DRs was reviewed by those who administered the questionnaires and, in cases of inconsistency or doubt about what was reported, data were verified directly with participants (Figure 1).

At the end of the study (one year later), nutrition professionals collected weight and a second FFQ. Lipid profiles and blood carotenoids were also evaluated. We used plasma carotenoids because they can be reliable biomarker of usual fruit and vegetable intake [20].

For this purpose, fasting blood samples were collected by antecubital vein puncture in tubes containing anticoagulant (EDTA). The samples were subsequently centrifuged (CIVEQ model 80-2 centrifuge) and the serum was stored at −70 °C (So-Low upright ultra-low temperature freezer, model 2-8254) until the time they were transported to the laboratory for analysis. For these samples, carotene was determined by chromatography while lipid profiles (total cholesterol, triglycerides, HDL, and LDL) were obtained via the enzymatic method.

### 2.2. Design and Calculation of the ICDMx

The calculation of ICDMx was based on energy and nutrient consumption results (unit/day) and food frequency intakes, taken from averages obtained from DRs and each FFQ that had been administered. This calculation also considered anthropometric data as well as data related to participants’ physical activity in order to determinate energy requirements.

The ICDMx was constructed based on recommendations for food and nutrient intake issued by Mexican experts (in line with international recommendations). These recommendations, as in other countries, consider intake needs as well as epidemiological evidence. This paper explains how the ICDMx was designed to assign scores that are higher or lower depending on the extent to which food consumption meets intake recommendations.

The ICDMx comprises five categories based on the National guideline [11]. Each category evaluates one of the following characteristics of a healthy diet: sufficient, balanced, complete, varied, and innocuous (Table 1). Based on scores from each category (maximum of 20 points), a total ICDMx score is obtained (maximum 100 points). While the concept of healthy diet [11] also includes the characteristic of “adequate diet”, ICDMx does not consider it because it is an indicator that includes subjective variables for each individual. In this case, according to the National guideline [11], “adequate” means that it is according with the preferences and culture of the population, and that it is adjusted to their economic resources, without sacrificing other characteristics of a healthy diet. This situation falls outside the scope of this study, which is focused on quantifying nutrients and food consumed based on established recommendations.

The determination of scores for each component is described in Table 1.

Component 1. Sufficient.

A rating of sufficient considers daily intake of energy, iron, calcium, fiber and water. In order to determine the score for energy, a maximum value (8 points) is assigned when intake covers 100% of the requirements [21] within a ±10% range. To calculate energy requirements, we considered the following variables: sex, age, weight, height and level of physical activity, based on national guidelines [21].

The cut-off points to determine the maximum scores (3 points) for iron [22], calcium [23], and fiber [24] are when these nutrients in the diet are ≥100% of the intake recommendations (IR).

The amount of bottled/filtered natural water consumed was classified considering recommendations for the Mexican population [25]. Three points are obtained when at least 1500 mL/day are consumed.

Component 2. Balanced.

This component considers consumption percentages (in kilocalories) of proteins, lipids and carbohydrates in comparison with total daily energy intake (this total energy intake includes kilocalories from alcohol). 

To determine the score for every aspect of this component, it is considered that if the consumed kilocalories of the macronutrient are within the recommended distribution percentages with respect to total kilocalories [26]. This category gets the maximum score: 7 points for proteins, when the distribution percentages are in the 12%–15% range [26]; 7 for lipids, when they are in the 25% to <30% [26,27] range; and 6 for carbohydrates, when they are in the 55%–63% range [26,28]. 

Component 3. Complete.

This component considers the consumption of the three food groups named in the Mexican *plato del bien comer* (plate of right eating) [11] guide. The maximum score is assigned when the following minimum number of recommended servings or grams of each food group are consumed daily: 400 g of vegetables and fruits [29], 200 g of cereals, and 120 g of legumes and animal products. For the latter two groups, the amounts needed to achieve a balanced diet for an individual with a total energy expenditure of at least 1550 kilocalories were considered [21], with the idea that a minority of the adult population would have lower requirements. 

Component 4. Variety

The ICDMx score for varied diet assumes that different foods from each of the three groups are consumed daily. To determine the scores for each food group, foods were first arranged into subcategories representing the respective groups. For the group of vegetables and fruits, five subgroups were identified based on recommendations from the *cinco por día* (five per day) program [30], in accordance with their predominant phytochemical content. Under this program, more than 5000 phytochemicals were identified, but the most studied were vegetables and fruits in five colors: red (lycopene and anthocyanins), bluish-purple (anthocyanins and phenolic compounds), yellowish-orange (vitamin C, carotenoids and bioflavonoids), green (lutein, zeaxanthin, glucosinolates and indoles), and white (allicin). To assign the maximum score for this subcomponent (8 points), it was determined that if at least four different subgroups of vegetables and fruits are consumed per day. For cereals, five subgroups were defined (wheat, rice, corn, whole grains, and tubers) such that if at least three different types of cereals were consumed, 6 points were awarded. In the subgroups of wheat and rice, we included only those that are not whole-grain. Those that are whole-grain were included in the whole-grain subgroup. For legumes and animal products, five subgroups (legumes, poultry -including eggs-, red meat -beef and pork-, seafood, dairy products), were included so that that if at least three different types of legumes or animal products were consumed, 6 points were awarded. 

Component 5. Innocuous.

This component measures moderation in the intake of foods or nutrients whose overconsumption often harms health. Food safety, that is also contained in National guideline [11], was not taken into consideration in terms of hygiene or toxicity because assessment of the latter category requires many more variables involving conditions of growth, transport, storage, and handling of foods that individuals who consume them are usually unaware of. The cut-off points to determine the maximum scores (5 points) for saturated fats and polyunsaturated fats were ≤7% and 6%–10% of energy intake respectively [27].

The cut-off points to determine the maximum scores for sodium and for alcohol (5 points) were ≤1600 mg/day [31] and ≤14.4 g of ethanol (equivalent to 1 alcoholic drink/day) [32], respectively. These alcohol cut-off points were based on lowest cut-off point in recommendations by the Academy of Nutrition and Dietetics [32]. In Mexico, recommended maximum consumption levels for alcohol have not been established.

### 2.3. Statistical Analysis

The calculation of the ICDMx was conducted from nutritional analysis, based on the algorithm of Table 1, in collaboration with the company Nutricloud^®^ (Guadalajara, Mexico), who developed a software for automated calculation. The average total ICDMx score (and standard deviation) was calculated from its components (characteristics of healthy diet) and its subcomponents (items evaluated for each characteristic) within the population. Reproducibility was measured by the Pearson linear correlation, which evaluates the correlation between variables, and intraclass correlation coefficients (ICC), which assesses agreement between methods [33], for both ICDMx indices for each component, subcomponent and the total score. Relative validity was assessed using the Pearson linear correlation coefficient and the ICC between the ICDMx2 and the ICDMxDR. Disattenuation of correlation coefficients caused by intra-individual variability was monitored using standard techniques [34].

Bland-Altman graphical tools [35] were used to enhance the understanding of validity results and identify deviation patterns for the total ICDMx score and for those of its components (this tool shows visually the agreement between methods). Linear regression analysis was performed to test if the slope of the mean bias was significantly different from zero. The agreement between methods (exact percentage agreement for tertile allocation in ICDMx2 v. ICDMxDR and ICDMx2 v. ICDMx1) was evaluated by comparing tertiles with crosstables, and Cohen’s κ coefficients were calculated. 

The validity analysis also determined the correlation between the total ICDMx score from FFQs and DRs, with serum biochemical and nutrient measurements obtained from the DRs using a Pearson correlation. Additional analysis compared ICDMx1, ICDMx2, and ICDMxRD with nutrients derived from diet records using a Pearson correlation. These nutrients were adjusted for energy intake using the residual method and logarithmic transformation was performed to provide normality (log 10) [36].

DRs, FFQs, and the ICDMx indices were processed using Nutricloud^®^ software; the SPSS program (version 17, SPSS Inc., Chicago, IL, USA, 2006) was used for statistical analyses while Medcalc software (Version 12.3.0.0, MedCalc Software, Mariakerke, Belgium) was used to generate Bland-Altman plots.

## 3. Results

The study examined 97 adults of 18–60 years of age (59 women and 38 men) who completed dietary questionnaires administered to them. A comparison was made of descriptive data for age, gender, occupation, marital status, level of physical activity, education, and BMI of those who completed the study and those who did not, with no significant differences having been found between them (Table 2). The average age of those who completed the study (*n* = 97) was 27.5 (SD 12.6) and the average BMI was 24.1 kg/m^2^ (SD 4.1). Most of the participants were single (82.5%), students (74.2%; 61.8% of the total number of participants were health science students), the largest majority of whom (44.3%) reported a sedentary level of physical activity and 94.8% reported ≥10 years of education (Table 2).

Table 3 shows the average scores for the ICDMx and its components as reported by each dietary questionnaire. The mean total value for the ICDMx (100 point maximum) for the population studied was: 65.8 (SD 9.3) for ICDMx1, 64.7 (SD 10.0) for ICDMx2 and 62.9 (SD 10.2) for ICDMxDR. The component with the highest score was ‘Complete’ (maximum 20 points), with an average of 18.1 (SD 1.9) for ICDMx1, 18.6 (SD 2.1), and 16.6 for ICDMx2 (SD 3.1) for ICDMxDR. The component with the lowest score was ‘Balanced’ (maximum 20 points), with an average of 8.6 (SD 5.4) for ICDMx1, 7.8 (SD 5.5) for ICDMx2 and 9.0 (SD 4.9) for ICDMxDR. The components ‘Sufficient’ and ‘Innocuous’ and their respective subcomponents obtained slightly higher scores for ICDMxDR, unlike the components ‘Complete’ and ‘Varied’, which obtained higher scores for ICDMx1 and ICDMx2. Regarding the differences among all the subcomponents in the ICDMx, among the three indexes calculated (ICDMx1, ICDMx2, and ICDMxRD), we observed minor differences for polyunsaturated fatty acids for the component Innocuous as there was a greater discrepancy for ‘Varied’ in the category of vegetables and fruits.

The comparative analysis between the ICDMx scores obtained by health science students (*n* = 60) and the general population (*n* = 37) showed no significant differences (*p* > 0.05). The mean total ICDMx score for health science students was ICDMx1 = 66.7 (SD 9.0), ICDMx2 = 65.3 (SD 10.5), and ICDMxDR = 63.5 (SD 10.0). Likewise, scores for the general population were as follows: ICDMx1 = 64.5 (SD 9.9), ICDMx2 = 63.7 (SD 9.1) and ICDMxDR = 61.8 (SD 10.4).

### 3.1. Reproducibility of the ICDMx

The reproducibility analysis for the total ICDMx score produced an ICC of 0.55 (Table 3), while the ICC for all five components ranged from 0.52 to 0.67 (mean = 0.62). All correlations for components, sub-components and the ICDMx total score were significant (*p* < 0.05), except in the case of the distribution of lipids sub-component.

The dietary component with the highest ICC was ‘Complete’ (ICC = 0.67). The ICC of its three subcomponents ranged from 0.54 for vegetables and fruits to 0.74 for legumes and animal products.

The component with the lowest correlation was ‘Balanced’ (ICC = 0.52). The ICCs of its three subcomponents ranged from 0.23 (not significant, NS) for the distribution of lipids to 0.60 for the distribution of carbohydrates.

The ‘Varied’ and ‘Innocuous’ components obtained an ICC of 0.66. The ICC of the three subcomponents of ‘Varied’ ranged from 0.56, due to variety in cereals, to 0.65, due to variety in legumes and animal products. The ICC of the four subcomponents for ‘Innocuous’ fell between 0.46 for polyunsaturated fatty acids, and 0.87 for alcohol.

Reproducibility for the component ‘Sufficient’ was ICC = 0.57. The ICC of its five subcomponents ranged from 0.57 for the energy adequacy ratio, to 0.74 for the calcium adequacy ratio.

### 3.2. Relative Validity of ICDMx

The validity analysis with DRs for the total ICDMx score obtained an ICC of 0.35 (Table 3), while the ICC for each of the five components ranged from 0.08 (NS) to 0.57. Most correlations for components, subcomponents and the total ICDMx score were significant (18/24; *p* < 0.05), except for the components ‘Balanced’ and ‘Complete’ and the subcomponents energy adequacy, lipid distribution, and intake of legumes and animal products and polyunsaturated fatty acids.

The components ‘Sufficient’ and ‘Varied’ obtained an ICC of 0.28. The ICC of four subcomponents of five, according to ‘Sufficient’, ranged from 0.42 for fiber adequate to 0.63 for water intake, and there was no correlation with energy adequacy (−0.06 (NS)). The ICC of the three subcomponents of ‘Varied’ ranged from 0.35 due to variety of cereals, legumes and animal products, to 0.47 due to variety of legumes and fruit.

The component Complete obtained an ICC of 0.22 (NS) for validity. The ICC of its three subcomponents ranged from 0.36 fruits and vegetables to 0.50 for cereal intake. There was no correlation with intake of “legumes and animal products” (0.22; NS).

**Figure 2:**
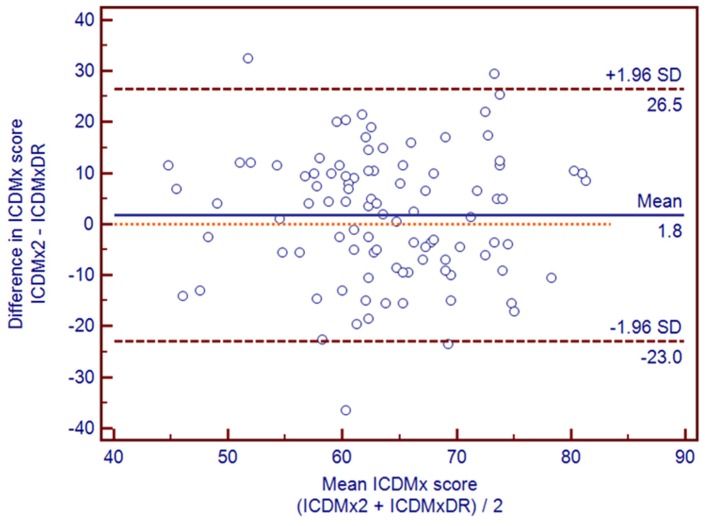
Bland-Altman plots illustrating the relationship between ICDMx score estimated from FFQ2 and the average ICDMx from nine-day DR (*n* = 97).

Figure 2 illustrates the Bland-Altman plot for the total ICDMx score. This figure illustrated a high level of agreement between methods. The total score for ICDMx2 tended to overestimate the total score of ICDMxDR by an average of 1.8 points (/100). The ICDMx2 score ranged from 26.5 points above and 23 below the total ICDMxDR score. The slope of this bias was not significantly different from zero (*p* = 0.65) in the linear regression analysis with the differences between methods as dependent variable and the means of the methods as independent variable. This means that the bias was consistent across scores ICDMx. In terms of the total value of the ICDMx and of each of its components, scores fell mostly within the limits of agreement (±1.96 SD).

The agreement was observed in 53.6% of cases, where the ICDMx2 and ICDMx1 placed an individual in the same tertile. The Cohen’s κ value suggests a fair agreement [37], statistically significant (κ = 0.304, *p* < 0.001). The degree of agreement between the ICDMx2 and ICDMxDR was similar with 52.6% (κ = 0.288, *p* < 0.001).

The other complementary validation process shows that the ICDMx1 score was inversely correlated with respect to total cholesterol (*r* = −0.33, *p* = 0.039) as was the ICDMx1 and ICDMx2 score for triglycerides (*r* = −0.22, *p* = 0.056 and *r* = −0.22, *p* = 0.053, respectively). Noteworthy in the correlation analysis of the scores calculated for the three ICDMx indices (ICDMx1, ICDMx2, and ICDMxDR, respectively) with nutrients calculated from DRs, because they were observed for all three tools, were direct correlations (*p* < 0.05) with fiber (*r* = 0.42, *r* = 0.23 and *r* = 0.60), magnesium (*r* = 0.27, *r* = 0.25 and *r* = 0.48), potassium (*r* = 0.30, *r* = 0.21 and *r* = 0.50), retinol (*r* = 0.43, *r* = 0.34 and *r* = 0.33), thiamine (*r* = 0.30, *r* = 0.21 and *r* = 0.24), riboflavin (*r* = 0.36, r = 0.29 and *r* = 0.19, considering that for the last value *p* = 0.06), pyridoxine (*r* = 0.29, *r* = 0.22 and *r* = 0.24), and folate (*r* = 0.44, *r* = 0.33 and *r* = 0.35).

## 4. Discussion

This article describes the rationale and evaluates the reproducibility and relative validity of a dietary quality index for the Mexican population (ICDMx) based on the assessment of the characteristics of a healthy diet [11] from a validated FFQ (FFQ-Mx) [18]. The ICDMx demonstrated adequate and significant reproducibility coefficients (*p* < 0.05) as shown by the scores for its components (ICC = 0.52–0.67) and by its total score (ICC = 0.55). Of the index’s 19 subcomponents, 11 obtained reasonably high ICC scores (0.60–0.87), considering that correlations have generally ranged 0.5 to 0.7, in validation studies of dietary surveys [19]: adequate levels of iron, calcium, and fiber; water intake; distribution of carbohydrates; intake of cereals, legumes and animal products; variety of legumes and animal products; intake of saturated fatty acids, sodium and alcohol. Similar results were obtained in a previous study, developed with a similar methodology and carried out on health professionals from the United States (US) [17], where the Revised Diet Quality Index (DQI-R) obtained adequate reproducibility for most of its components (*r* = 0.41–0.76). In the DQI-R, the components that demonstrated the highest levels of reproducibility were: dietary variety, fruit intake, and calcium intake. A more recent study identified that the reproducibility coefficient of Australian Recommended Food Score was ICC = 0.87 (95% CI 0.83, 0.90) [10]. However, some differences with respect to the present study are observed in the methodology, specifically the time interval between applications (five months). In addition, the age of Australian participants is older than in the present study (median >40 years old).

The ICDMx obtained significant relative validity coefficients (*p* < 0.05) for three of its five components (ICC = 0.28–0.57) and for its total score (ICC = 0.35). Of its 19 subcomponents, only 4 were NS. Although the ICDMx did not obtain a significant ICC for all items, it is considered appropriate to include all of them in the calculation of ICDMx because it is part of the “healthy diet” concept expressed in Mexico’s food guide [11]. Moreover, the low ICC and the ICC no significant scores could be attributable to the fact that the comparison methodology used is not a perfect “gold standard”. The above-mentioned sources of error of DRs [19] mean that some aspects, such as dietary variety may have been underreported, the tendency to simplify dietary intake or underreport foods like oil or salt, if the subject has not been adequately trained. However, in this study DRs were chosen because they are the tool that has shown the least correlated errors, among the comparison methods available and feasible for evaluating dietary intake [19] and because they minimize recall and food portion perception errors to a greater degree than methods such as 24-h recall. Meanwhile, the FFQ has potential sources of error as: the limitations inherent to include a fixed list of foods, memory errors, or the limited ability of subjects to perceive the portion sizes indicated in the FFQ. In addition, to minimize the source of error of DRs, a standardization of interviewers was carried out to ensure that they trained participants on the proper ways to report their food intake. 

Because there is no perfect gold standard to compare dietary surveys, these studies could strictly be called inter-method reliability. However, studies comparing with a superior method have used the term validity [7,8,9,15,17,19], or more specifically: relative validity [5] or comparative validity [10]. Based on this, and the definition of validity for dietary surveys [19], the present study uses the term relative validity.

The ranking of correlations in this research was similar to that reported in other studies (ICC = 0.06–0.72), including one conducted on a Spanish population [7], another carried out on a Malian population [8] and another on a population in the US [17]. However, the total score for the DQI-R from the US obtained a considerably higher correlation (*r* = 0.73). The ICDMx subcomponents with the best validity correlations were alcohol, water, and saturated fatty acid intake (ICC = 0.61–0.79), two of which were part of the component Innocuous. Similar results were obtained by DQI-R [17], where the correlation for the component Moderation was high (*r* = 0.68).

The main difference between these studies is the food guide used, as each one considers the guidelines established for its population. Moreover, the number of days that DRs were evaluated to calculate the comparison index also varied. The number of days were 2 [8], 12 [7], and 14 [17], compared to 9 days for the ICDMx. These periods were considered sufficient to reflect seasonal variations and variations between working and non-working days. Another difference was the minimum and maximum scores for dietary quality indices. While the ICDMx coincides with the DQI-R in terms of scoring between 0 and 100, the same does not occur with the Mediterranean Diet Scores (MDS), whose values range between 13 and 39 [7], or that validated in the Malian population where, instead of being given a score, they were classified based on the percentages of adequacy of some nutrients [8].

The Bland-Altman plots showed a good level of agreement between methods (ICDMx2 vs. ICDMxDR). A slight tendency to overestimate the ICDMx2 compared to the ICDMxDR by an average of 1.8 points (/100) was observed. The score of the ICDMx2 varied by 26.5 points (/100) above and 23 points below the total ICDMxDR score. Lower score differences are desirable for the limits of agreement (represented by values ± SD 1.96), because this would indicate greater agreement when calculating the ICDMx from both methods (2nd FFQ and DR). However, considering the changes in the intake of foods that naturally are observed in a year, and that the ICDMx has a range between zero and 100, the values of the limits of agreement (+26.5 and −23) are reasonable and even show that there is no systematic error, because they are distributed in a similar way, upwardly and below the line that is representing a difference of zero. In comparative terms, in a previous study on parents of children aged 4–11 [5], the Bland-Altman plots reported an average overestimation of the Dietary Guideline Index for Children and Adolescents (DGI-CA) obtained by FFQ of 16 points (/100). In addition, the score of this DGI-CA by FFQ ranged from 39 points (/100) above and 7 below. 

On the other hand, the total score of the ICDMx and its five components showed that most of its findings were within the limits of agreement. Moreover, no cases showed a tendency for the degree of disagreement to increase or decrease when the score obtained for a particular item increased, a fact which coincides with the findings of MDS [7] and DGI-CA [5]. 

Bland-Altman plots illustrated a high level of agreement between methods; ICDMx scores were inversely correlated with respect to total cholesterol (*r* = −0.33, *p* = 0.039 with ICDMx1) and triglycerides (*r* = −0.22, *p* = 0.056 with ICDMx1 and *p* = 0.53 with ICDMx2). This implies that lower cholesterol and triglyceride levels were detected in higher quality diets. For its part, the DQI-R from US [17] showed an inverse correlation with total cholesterol (*r* = −0.22, *p* < 0.05) and a direct correlation with five carotenoids (*r* = 0.17–0.43, *p* < 0.05). Another study on women in the US with an average age of 53.7 years [9] and whose purpose was to evaluate nutritional risk, found inverse correlations between the component of vegetables and fruits from the Dietary Risk Assessment Index (DRAI) and blood determinations of four carotenoids (*r* = −0.15 to −0.35, *p* < 0.05). The Food Frequency Index (FFI), validated for Austrian men and women with an average age of 75 [3] identified correlations with vitamin D (*r* = 0.2, *p* < 0.05), HDL cholesterol (*r* = 0.24, *p* < 0.05) and four carotenoids (*r* = 0.19 to 0.31, *p* < 0.05), but not with total cholesterol (*r* = 0.04, *p* > 0.05). Of interest is the similarity of results for components with a lipid profile, but not the comparison with carotenoids. This may be because, in this study, they were assessed as a whole and not separately as in the DQI-R [17], the DRAI [9], and the FFI [3].

ICDMx scores were also compared to the nutrients obtained from DRs. Scores calculated by the three ICDMx indices (ICDMx1, ICDMx2, and ICDMxDR) were correlated directly with fiber, magnesium, potassium, retinol, thiamin, riboflavin, pyridoxine, and folate. Noteworthy is the observation that these nutrients (except fiber) are not directly included in the calculation of the ICDMx, although they can be provided by some food groups. So, even if they could reflect food consumption, the correlation with this calculated score suggest, as Newby and colleagues [17], that the ICDMx captures additional aspects of diet quality that are not explicitly involved in this score calculation. 

One strength of this study is that the ICDMx is the first instrument to assess dietary quality among the Mexican population based on national dietary guidelines [11] and that report reproducibility and relative validity results. A previous study on the Mexican population developed three indices to assess dietary quality among Mexican adults: a Cardioprotective Index, a Micronutrient Adequacy Index, and a Dietary Diversity Index [12]. However, these studies focused on the assessment of certain aspects of diet and did not include all key elements of a healthy diet, as proposed in the national food guide [11] in consideration of nutritional recommendations for the Mexican population [21,22,23,24,25,26,27,28,29,30,31]. Meanwhile, the previous study [12] assessed the discrimination capacity of dietary quality indices across the board, based on the results of a national survey, but it did not evaluate their reproducibility or validity.

The assessment of reproducibility of the ICDMx one year after its introduction demonstrates the overall coherence of the ICDMx, assuming the possibility of true changes in dietary intake during the year (due to real changes in dietary intake, a higher reproducibility was thus not expected). The relative validity of the ICDMx shows its capacity to assess the quality of habitual diet using a FFQ (in comparison to standard evaluations of diet during nine days in a year). Future studies should analyze associations between ICDMx results and chronic diseases. Likewise, the use of different methods for measuring validity provides a more complete picture with implications for future implementation. The ICCs reported the correlation between the data obtained from a validated FFQ (FFQ-Mx) [18] compared with DRs, which is considered as the most commonly used benchmark method. Moreover, the Bland-Altman plots showed agreement between methods, and also the comparison of the ICDMx with biochemical parameters and nutrients from the DRs. They complemented validation by showing the direction and magnitude of correlations with these indicators. 

The homogeneity of the subjects is a possible limitation of the study, as most subjects were students of health sciences. Within the descriptive data of the ICDMx, average total scores that could be interpreted as being low were observed with regard to this aspect (ICDMx1 = 65.8, ICDMx2 = 64.7, and ICDMxDR = 62.9). However, similar results were found in a study on health professionals (DQI-R1 = 69.6, DQI-R2 = 67.2, and DQI-R-RD = 62.0) [17]. Likewise, results in another study also provide an index score between 0 and 100, with data taken from a national survey of the general population and which reported dietary quality averages between 61.4 and 63.8 [6]. Further studies should aim to validate the ICDMx in population with different characteristics of age, academic education, and socioeconomic status, and maybe, with a continuous scoring algorithm we might provide more precise estimates of individual diet quality, and then, determine with precision ICDMx differences between populations, with consideration of these factors.

The ICDMx is a tool based on nutrients and food groups, and requires a prior analysis of the FFQ. This could be a limitation compared to other indices that allow for more rapid dietary assessment. However, this can be a strength in studies that aim to make a more accurate assessment, considering these elements.

## 5. Conclusions

The ICDMx is a tool for the assessment of habitual diet in accordance with Mexican criteria for a healthy diet, in the study population, which has demonstrated acceptable levels of reproducibility and relative validity. It will be useful in epidemiological studies, with similar population, for classification according to the quality of their diet and for the analysis of associations with diseases for which diet is a risk factor. Moreover, it will facilitate diagnoses and the assessment of changes resulting from the implementation of nutritional interventions.

## Figures and Tables

**Table 1:** ICDMx components and their relationship to the key recommendations of the daily dietary guide (unit/day) for the Mexican population (maximum: 100 points).

Daily Recommendation: Healthy Diet [11]	Components of the ICDMx (Maximum: 100 Points)	Scoring Criteria
**Component 1. Sufficient (20 points)**		
Meets all nutritional needs, so that the adult subject has good nutrition and a healthy weight.	The diet covers 100% of energy requirements [21].	>90% or <110% = 8
		>80%–90% or 110%–<120% = 7
		>70%–80% or 120%–<130% = 6
		>60%–70% or 130%–<140% = 5
		>50%–60% or 140%–<150% = 4
		>40%–50% or 150%–<160% = 3
		>30%–40% or 160%–<170% = 2
		>20%–30% or 170%–<180% = 1
		≤20 or ≥180% = 0
	Iron intake of at least 21 mg for women or 15 mg for men [22], % RDA for age.	≥100% = 3
		50%–<100% = 1.5
		<50% = 0
	Calcium intake of at least 1 g [23], % AI for age.	≥100% = 3
		50%–<100% = 1.5
		<50% = 0
	Fiber intake of at least 30 g for women or 35 g for men [24], % nutritional recommendations.	≥100% = 3
		50%–<100% = 1.5
		<50% = 0
	Water intake of at least 1500 mL [25].	≥1500 = 3
		1000–<1500 = 1.5
		<1000 = 0
**Component 2. Balanced (20 points)**		
Nutrients are consumed in balanced proportions with respect to total energy intake.	Proteins, 12%–15% of energy intake [26].	12%–15% = 7
		10–<12% or >15%–19% = 3.5
		<10 or >19% = 0
	Lipids, 25%–<30% of energy intake [26,27].	25%–<30% = 7
		21%–<25% or 30%–33% = 3.5
		<21% or >33% = 0
	Carbohydrates, 55%–63% of energy intake [26,28].	55%–63% = 6
		51%–<55% or >63%–67% = 3
		<51% or >67% = 0
**Component 3. Complete (20 points)**		
Contains all nutrients. It is recommended to include foods from all three groups in each meal.	It includes at least 400 g from the vegetables and fruits group [29].	≥400 = 8
		300–<400 = 6
		200–<300 = 3
		<200 = 0
	It includes at least 200 g from the cereals group.	≥200 = 6
		150–<200 = 4
		100–<150 = 2
		<100 = 0
	It includes at least 120 g from the legumes and animal product group.	≥120 = 6
		90–<120 = 4
		60–<90 = 2
		<60 = 0
**Component 4. Varied (20 points)**		
Different foods from each group are consumed. This implies a variety of textures, colors, flavors, etc.	It includes at least four of five sub-groups (red, bluish-purple, yellowish-orange, green and white) from the vegetables and fruits group [30].	4 or 5 = 8
		3 = 6
		2 = 3
		<2 = 0
	It includes at least three of five sub-groups (wheat, rice, corn, whole grains and tubers) from the cereals group.	3–5 = 6
		2 = 3
		<2 = 0
	It includes at least three of five sub-groups (legumes, poultry -including eggs-, red meat -beef and pork-, seafood, dairy products) from the legumes and animal product group.	3–5 = 6
		2 = 3
		<2 = 0
**Component 5. Innocuous (20 points)**		
Its regular intake does not entail health risks because it is consumed in moderation.	Saturated fatty acids, ≤7% of energy intake [27].	≤7% = 5
		>7%–12% = 2.5
		>12% = 0
	Polyunsaturated fatty acids, 6%–10% of energy [27].	6%–10% = 5
		>10%–15% or <6% = 2.5
		>15% = 0
	Sodium intake, 1600 mg [31].	≤1600 = 5
		>1600–2600 = 2.5
		>2600 = 0
	Alcoholic drink intake, ≤14.4 g of ethanol (equivalent to 1 alcoholic drink) [32].	≤14.4 = 5
		>14.4–21.6 = 2.5
		>21.6 = 0

RDA, Recommended Dietary Allowance; AI, Adequate Intake.

**Table 2:** Characteristics of participants who completed the study (*n* = 97) and those who did not complete (*n* = 53).

	Participants (*n* = 97)		Non-Participants (*n* = 53)	
Characteristics	*n*	%	*n*	%
**Age ^1^**	27.5	12.6	24.5	7.9
**BMI ^1^**	24.1	4.1	24.2	3.1
**Gender ^2^**				
Male	38	39.2	28	52.8
Female	59	60.8	25	47.2
**Physical activity ^3^**				
Sedentary	43	44.3	27	50.9
Active	38	39.2	22	41.6
Very active	16	16.5	4	7.5
**Marital status ^3^**				
Single	80	82.5	50	94.3
Married	16	16.5	3	5.7
Divorced	1	1.0	0	0
**Education ^3^**				
<10 years	5	5.2	3	5.7
≥10 years	92	94.8	50	94.3
**Occupation ^3^**				
Employee	20	20.6	8	15.1
Homemaker	1	1.0	0	0
Student	72	74.2	45	84.9
Unemployed	4	4.1	0	0

^1^ This data are presented as mean and standard deviation, t test *p* > 0.05; ^2^ Chi square *p* > 0.05; ^3^ Fisher’s exact test *p* > 0.05.

**Table 3:** Comparison between the ICDMx calculated from the FFQ2, and the average from DRs (*n* = 97).

Component/Subcomponent		ICDMx1		ICDMx2		ICDMxDR		Reproducibility *		Validity *	
	Maximum	Mean	SD	Mean	SD	Mean	SD	r	ICC	r	ICC
**Component 1 Sufficient**	20	**13.2**	2.9	**13.2**	2.7	**13.4**	2.7	0.40	**0.57**	0.16 ^†^	**0.28**
Adequate energy	8	**5.3**	2.7	**5.4**	2.2	**6.3**	1.4	0.41	**0.57**	−0.03 ^†^	**−0.06 ^†^**
Adequate iron	3	**2.4**	0.8	**2.3**	0.8	**2.1**	0.8	0.47	**0.63**	0.39	**0.57**
Adequate calcium	3	**2.4**	0.9	**2.2**	0.9	**1.9**	0.9	0.58	**0.74**	0.41	**0.59**
Adequate fiber	3	**1.5**	1.0	**1.4**	1.1	**1.1**	0.9	0.54	**0.69**	0.27	**0.42**
Water intake	3	**1.7**	1.0	**1.9**	1.0	**1.9**	1.2	0.50	**0.67**	0.45	**0.63**
**Component 2 Balanced**	20	**8.6**	5.4	**7.8**	5.5	**9.0**	4.9	0.37	**0.52**	0.01 ^†^	**0.08 ^†^**
Distribution of proteins	7	**4.8**	2.2	**4.7**	2.2	**3.0**	2.0	0.20	**0.33**	0.28	**0.45**
Distribution of lipids	7	**1.8**	2.6	**1.4**	2.4	**3.3**	2.7	0.11 ^†^	**0.23 ^†^**	−0.02 ^†^	**−0.15 ^†^**
Distribution of carbohydrates	6	**2.0**	2.5	**1.7**	2.2	**2.7**	2.5	0.43	**0.60**	0.19 ^†^	**0.33**
**Component 3 Complete**	20	**18.1**	1.9	**18.6**	2.1	**16.6**	3.1	0.52	**0.67**	0.14 ^†^	**0.22 ^†^**
Vegetables and fruits	8	**7.7**	1.0	**7.7**	1.2	**5.3**	2.9	0.38	**0.54**	0.31	**0.36**
Cereals	6	**5.3**	1.4	**5.0**	1.7	**5.6**	0.9	0.56	**0.71**	0.38	**0.50**
Legumes and AOF	6	**5.9**	0.3	**5.9**	0.4	**5.7**	0.9	0.58	**0.74**	0.17 ^†^	**0.22 ^†^**
**Component 4 Varied**	20	**14.1**	3.6	**13.4**	3.9	**10.7**	4.5	0.49	**0.66**	0.17 ^†^	**0.28**
Variety of vegetables and fruits	8	**6.4**	2.2	**6.3**	2.2	**2.7**	2.7	0.40	**0.57**	0.31	**0.47**
Variety of cereals	6	**2.4**	2.1	**2.3**	2.0	**3.2**	2.2	0.39	**0.56**	0.21	**0.35**
Variety of legumes and AOF	6	**5.3**	1.4	**4.8**	2.0	**4.7**	1.9	0.51	**0.65**	0.21	**0.35**
**Component 5 Innocuous**	20	**10.8**	3.9	**11.6**	3.4	**13.1**	2.9	0.50	**0.66**	0.39	**0.57**
SFA	5	**1.2**	1.5	**1.6**	1.6	**2.8**	1.6	0.61	**0.76**	0.43	**0.61**
PUFA	5	**3.3**	1.6	**3.3**	1.4	**3.3**	1.2	0.30	**0.46**	0.05 ^†^	**0.08 ^†^**
Sodium	5	**1.9**	2.0	**2.1**	1.9	**2.3**	1.7	0.57	**0.72**	0.12 ^†^	**0.26**
Alcohol	5	**4.5**	1.5	**4.6**	1.2	**4.8**	0.9	0.78	**0.87**	0.65	**0.79**
Total score of the ICDMx	100	**65.8**	9.3	**64.7**	10.0	**62.9**	10.2	0.39	**0.55**	0.19	**0.35**

ICDMx, *Índice de Calidad de la Dieta Mexicana*; FFQ, semi-quantitative food frequency questionnaire; DRs, dietary records; ICDMx 1, ICDMx from basal FFQ; ICDMx2, ICDMx from second FFQ (one year later); ICDMxDR, ICDMx from DRs; r, Pearson linear correlation coefficients; ICC, interclass disattenuated correlation coefficients; AOF, animal products; * All correlations were significant (*p* < 0.05), except those marked with ^†^.

## Abbreviations

The following abbreviations are used in this manuscript:

BMI	Body Mass Index
DGI-CA	Dietary Guideline Index for Children and Adolescents
DQI-R	Revised Diet Quality Index
DRAI	Dietary Risk Assessment Index
DRs	Dietary Records
FFI	Food Frequency Index
FFQ	Food Frequency Questionnaire
HDL	High Density Lipoprotein
ICC	Intraclass Correlation Coefficients
ICDMx	Mexican Diet Quality Index
ICDMx1	ICDMx from basal FFQ
ICDMx2	ICDMx from second FFQ (1 year later)
ICDMxDR	ICDMx from DRs
IR	Intake Recommendations
LDL	Low Density Lipoprotein
MDS	Mediterranean Diet Score
NS	Not significant
US	United States
